# Estimating Past, Present, and Future Trends in the Global Distribution and Abundance of the Arbovirus Vector *Aedes aegypti* Under Climate Change Scenarios

**DOI:** 10.3389/fpubh.2019.00148

**Published:** 2019-06-12

**Authors:** Jing Liu-Helmersson, Åke Brännström, Maquins Odhiambo Sewe, Jan C. Semenza, Joacim Rocklöv

**Affiliations:** ^1^Department of Epidemiology and Global Health, Umeå University, Umeå, Sweden; ^2^Department of Mathematics and Mathematical Statistics, Umeå University, Umeå, Sweden; ^3^Evolution and Ecology Program, International Institute for Applied Systems Analysis, Laxenburg, Austria; ^4^Department of Public Health and Clinical Medicine, Section of Sustainable Health, Umeå University, Umeå, Sweden; ^5^European Centre for Disease Prevention and Control, Stockholm, Sweden

**Keywords:** *Aedes aegypti*, mathematical model, vector abundance, temperature, precipitation, climate change, socioeconomic factors, global vector abundance

## Abstract

**Background:** *Aedes aegypti* is the principal vector for several important arbovirus diseases, including dengue, chikungunya, yellow fever, and Zika. While recent empirical research has attempted to identify the current global distribution of the vector, the seasonal, and longer-term dynamics of the mosquito in response to trends in climate, population, and economic development over the twentieth and the twenty-first century remains to be elucidated.

**Methods:** In this study, we use a process-based mathematical model to estimate global vector distribution and abundance. The model is based on the lifecycle of the vector and its dependence on climate, and the model sensitivity to socio-economic development is tested. Model parameters were generally empirically based, and the model was calibrated to global databases and time series of occurrence and abundance records. Climate data on temperature and rainfall were taken from CRU TS3.25 (1901–2015) and five global circulation models (CMIP5; 2006–2099) forced by a high-end (RCP8.5) and a low-end (RCP2.6) emission scenario. Socio-economic data on global GDP and human population density were from ISIMIP (1950–2099).

**Findings:** The change in the potential of global abundance in *A. aegypti* over the last century up to today is estimated to be an increase of 9.5% globally and a further increase of 20 or 30% by the end of this century under a low compared to a high carbon emission future, respectively. The largest increase has occurred in the last two decades, indicating a tipping point in climate-driven global abundance which will be stabilized at the earliest in the mid-twenty-first century. The realized abundance is estimated to be sensitive to socioeconomic development.

**Interpretation:** Our data indicate that climate change mitigation, i.e., following the Paris Agreement, could considerably help in suppressing risks of increased abundance and emergence of *A. aegypti* globally in the second half of the twenty-first century.

## Introduction

*Aedes aegypti* (L.) is the principal vector for many arboviral diseases, including dengue, chikungunya, yellow fever, and Zika (1–4). They have (re-) emerged in many densely populated, tropical and sub-tropical areas around the globe, in part due to global trade and travel (5–8). Almost half of the world's population is now at risk from these diseases (9).

*A. aegypti* is an ectotherm species, dependent on warm and humid conditions for reproduction and disease transmission. Despite these physiological constraints, it is not well-understood to what extent climate and climate change have contributed to the global expansion of *A. aegypti*. Moreover, it is not clear whether climatic factors have affected its abundance, critical for an epidemic threshold. Socio-economic factors have been found to correlate with the immature-stage population of *Aedes albopictus* mosquitos in the USA through variations in the number and the type of breeding sites, e.g., storage and disused containers (tires, food/drink containers) (10, 11). In general, the lower the neighborhood income, the higher the *Aedes* mosquito production (11). However, from malaria studies, socio-economic development is found to decrease the global distribution of malaria (12). The concurrent contributions of changes in climate and socio-economic development have been difficult to disentangle. A number of attempts have been made to map the spatial presence of two *Aedes* vectors (*A. aegypti* and *A. albopictus*) (13–16). However, despite these comprehensive efforts on its presence, vector abundance is still poorly understood. There is no spatial map on *Aedes'* vector abundance global distribution from either empirical data or from modeling. Neither can we find the vector's abundance seasonal patterns globally.

Statistical modeling has been used to estimate vector presence, for example, from Kraemer et al. (14) and Santos and Meneses (17). Among environmental and climatic covariates, climatic conditions were the most important predictors of vector presence in this model, ahead of the vegetation parameter (14). In contrast, urbanization accounted only for 2% of the variance, which is difficult to reconcile in light of large-scale domestication of *A. aegypti* and adaptation to urban settings (14, 18, 19). A possible explanation is that statistical niche models suffer from under-reporting and collection bias, inherent in even the most comprehensive global vector surveillance repository. Moreover, data on vector abundance are largely unavailable. Niche modeling is also affected, albeit indirectly, by socio-economic (e.g., land use) and public health factors (e.g., vector control activities), which in turn affect the presence/absence of *A. aegypti*.

In contrast, mathematical modeling has been used to predict vector population dynamics based on documented biological processes (rather than vector presence/absence data), the vector's lifecycle, breeding and survival rates, and the influence of external drivers for vector development (20–22). This vector development mechanism can be parametrized with empirical data from the laboratory or field. Such a mathematical model can be built on collectively gathered empirical evidence to estimate vector proliferation, and then combined with climatic, environmental, and socio-economic factors. By applying such a dynamic system to estimation of vector development and demography, the vector population can be described in spatio-temporal terms.

However, most prior researches in this field have not dynamically adjusted the parameters in space and time, to reflect naturally occurring changes in weather and climate. Instead they have retained constant parameters throughout (23), fixed seasonal variation (24), or considered mainly temperature with limited or no rainfall (7, 21, 22). Previous mathematical models have all focused on vector population dynamic for one or two specific areas only (7, 17, 21, 22). No study has described global vector distribution and abundance, past trends and future scenarios. In addition, socio-economic factors have not been considered in mathematical modeling of *A. aegypti* vector population dynamics. Our recent work has demonstrated that process-based mathematical modeling is a very good tool to study the vector infestation of new areas through its growth rate. We have shown how the climate change may affect the future infestation of *A. aegypti* in Europe (25).

In this study we use the same process-based mathematical model to estimate the global distribution and abundance of *A. aegypti*, in response to climatic variables and socio-economic factors. We validate this model against global vector presence data. Then we project the global distribution, the potential abundance, and the vector density from the past to the future, taking into account either climate as a sole factor or climate and socio-economic factors together. Future climate scenarios are used to project the potential vector abundance to the end of the twenty-first century.

## Method

We used a process-based mathematical model with three compartments based on the lifecycle of *A. aegypti*: Larvae, Pupae, and Adults (see Supplementary Material, S1) (20). Two models are used in this study: Model A uses only climate as the driving force (Figure S1A) to estimate the vector potential abundance; Model B uses climate, gross domestic product per capita (GDPpc), and human population as the driving forces (Figure S1B) to estimate vector density. The modeling framework, differential equations and model parameters used are described in the Supplementary Material. Potential vector abundance is defined as the average number of female vectors during a given time period that can be generated from each larval site, and is fully dependent on climatic conditions. The number of larval sites per square kilometer is estimated through human population density and GDPpc. From this, potential vector abundance is transformed to estimate the actual vector density.

All the mosquitoes' vital rates (birth, death, and transition) depend on temperature and/or precipitation (26). In addition, the egg-to-larva hatching fraction depends on the larval population and environmental carrying capacity, due to competition for survival (27). The whole lifecycle from egg to adult takes one to a few weeks on average, depending on temperature and precipitation (20). Those temperature dependent parameters are obtained from laboratory studies (28) and precipitation dependent parameters were modeled (20) and validated against local dengue outbreaks (25).

In Model A, the external variables include only climate forcing, temperature and rainfall, as the influencing factors to the vector's development, vital rates and environmental carrying capacity for larvae, assuming ideal situation for fecundity rate. Model B expands Model A through two terms: the fecundity rate and the environmental carrying capacity for larvae. A human blood meal factor is included in the fecundity rate to account for the blood meals needed for the mosquito's reproduction. A larval site density is included in the environmental carrying capacity for larvae to account for human contributions to the larval (breeding) sites. The larval site density involves two factors: human population density and GDPpc (see Figure S1B and Equation 1B), to simulate the human creation of extra larval breeding sites.

Climate data on monthly mean temperature and rainfall were obtained from CRU TS3.25 (1901–2016) (29) and ISIMIP (GCM, CMIP5 2006–2099) (30) for two future scenarios, RCP2.6 and RCP8.5. Socio-economic data on yearly global GDP and human population density were obtained from ISIMIP (1950–2099) (30) based on scenarios SSP2 (Shared Socioeconomic Pathways) (31). All data are gridded (0.5 ×0.5 degrees). Spline interpolation function was used to obtain continuous data before solving the equations. The parameters used in the model were documented and are described in Tables S1A,B. Programs *Wolfram Mathematica* 11 and *R* (package deSolve 1.20) (32) are used to obtain time series vector population and abundance values, and to generate global maps.

Humans contribute to the larval sites directly or indirectly through agriculture, polyculture, and urbanization (10, 11, 33), therefore increasing the environmental carrying capacity for larval development which is captured in Model B. In general, the higher the human population in a given area (ρ), the more the larval sites (*d*). The lower the socio-economic level, the more the water storage and waste containers that allow *Aedes* mosquito to develop. We assume that the number of larval sites *d* is proportional to the human population and inversely proportional to the economic level, which we approximate by the square root of the ratio of GDP/capita (*g*) to the world average value (*g*_*a*_)—see Equation (5) in Supplementary Material (Table S1B) for details. Human contribution to blood meals is accounted as an extra factor *h* (human blood-meal factor), which describes the possible reduction in oviposition rate outside laboratory (ideal) conditions.

The details are described in the Supplementary Material—how the variables or model parameters relate to weather, human population, and GDPpc (see S2). We also show the parameters' optimization by calibrating the model output of female *A. aegypti* adult population of Model B to the field study in Brazil [S3, (34)]. In addition, we have performed sensitivity analysis to test how the nine model parameters used affect the model output for six cities in the world using Model B (S4). Finally, we have validated our models through comparison of the global maps generated with the existing global occurrence data.

## Results

### Climate Driven Abundance Predictions

**Figure 1:**
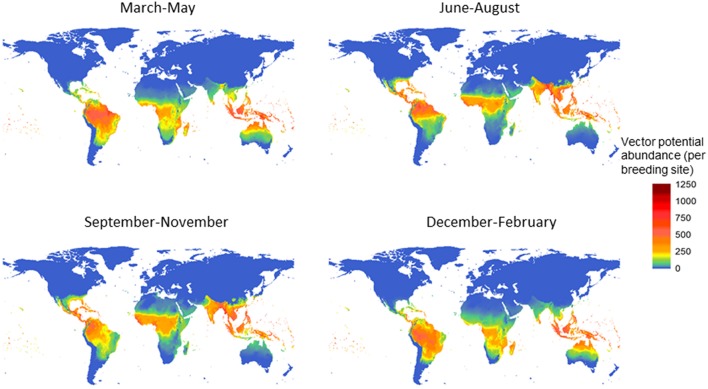
Potential for global distribution and abundance of female *Aedes aegypti* over the decade 2000–2009, by season. The vector population was first summarized for each season−3 months-and then averaged over the decade. The unit is vector population per larval site, and model A was used.

The potential for global distribution and abundance of female adult *A. aegypti* is estimated by season, based on climate variability alone (Figure 1; Model A). After introducing a small female adult population in the warmest season of the year in 1999, vector populations at each stage were simulated up to the end of 2009. The potential abundance of the female adult population was summarized for each season (December–February; March–May; June–August; September–November) and then averaged over one decade (2000–2009). The highest potential abundance of the female adult vector is estimated along the equator, in south Asia, mid - Africa, Central America, and most of South America (Figure 1). Among the four seasons, the summer and autumn—which correspond to June to November in the northern hemisphere, or December to May in the southern hemisphere—have the highest potential for vector abundance. This postdiction (Model A) showed high agreement when validated to a rich dataset of vector occurrence records (see Supplementary Material S5).

**Figure 2:**
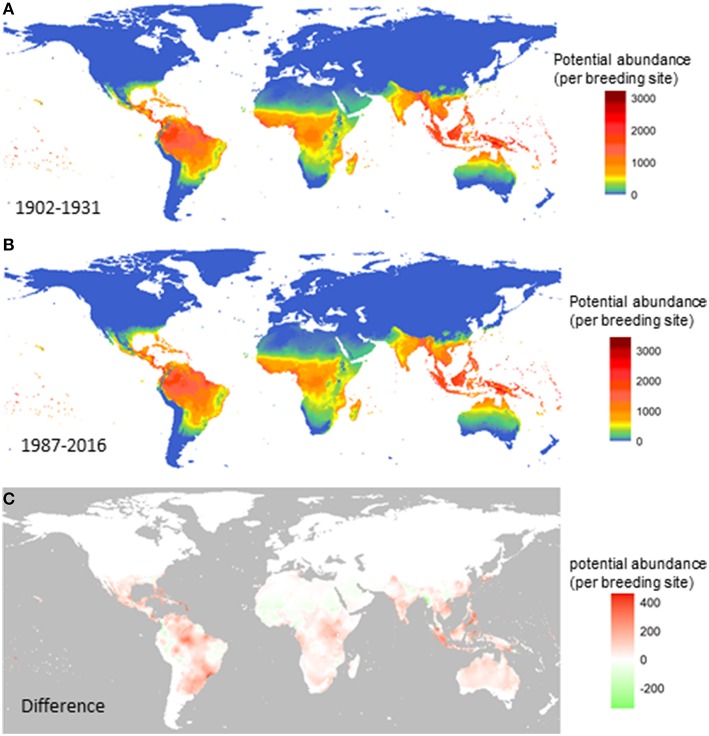
Change in the potential abundance of *Aedes aegypti* (per larval site) over the 20th century. The female vector population was first summarized for each year and then averaged over three decades at the beginning of the last century **(A)**, the turn of this century **(B)**. The difference between **(B)** and **(A)** is shown in **(C)**. Model A was used.

Next, we compare the potential for global distribution and abundance of *A. aegypti* over the past century. Figure 2 shows the yearly averaged abundance (total female adults per larval site) over the three decades at the beginning of the last century (1902–1931; Figure 2A) and at the turn of this century (1987–2016; Figure 2B), and the difference between the two (Figure 2C). Changes are observed along the tropical belt, and increased potential for abundance (red) is postdicted in Central and South America, especially in Mexico, Cuba, Venezuela and parts of Brazil (north, northeast, southeast coastal states from Rio de Janeiro, San Paulo to Rio Grande do Sul); in central Africa around the Uganda and Kenya area; in South Asia including Indonesia, Singapore, Malaysia, Vietnam, and the Philippines. Decreased potential for vector abundance is postdicted in only a few areas (green), including scattered small areas in the west and east part of South America, in north Africa, and in a smaller strip from Nepal to the west coast of Myanmar.

**Figure 3:**
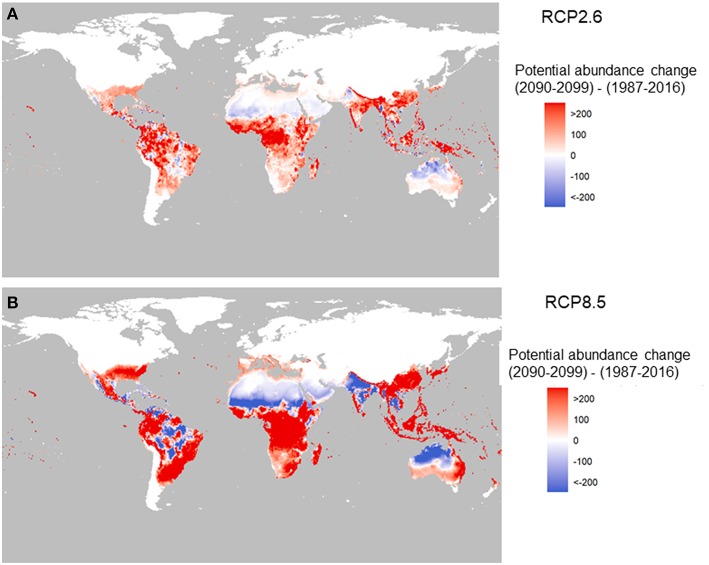
Change in the potential abundance of *Aedes aegypti* (per larval site) over the twenty-first century (2090–2099 relative to 1987–2016). The two panels correspond to two carbon emission scenarios: RCP2.6 **(A)** and RCP8.5 **(B)** using Model A.

The future change in potential for global abundance is shown in Figure 3 for two RCPs and is based on the average of 5 global-circulation model (GCM; CMIP5) at the 0.5 ×0.5 arc degree global grid scale. Here the change means the difference in total female adults per larval site per year between 2090–2099 and 1987–2016. We have chosen the global abundance value during 1987–2016 as the baseline to see the changes in the end of this century relative to the beginning of this century. This is plotted with red representing for increase and blue for decrease.

Under RCP2.6, compared to under RCP8.5 (Figures 3A,B) there is a stark contrast in the potential abundance of the vector. The low-emission scenario pathway, RCP2.6, indicates positive changes in Asia such as in China, and central and West Africa, and Latin America, but reductions in India and Australia. The high-emission scenario, RCP8.5, shows big increased potential in Southeast Asia, China, Japan, East Australia, and Africa (with the exception of the Sahel area). In the Americas, the change is the greatest in highly populated areas of Brazil, Mexico and the US, with drops in the abundance potential in central South America, north Africa, southwest Asia, and North Australia.

**Figure 4:**
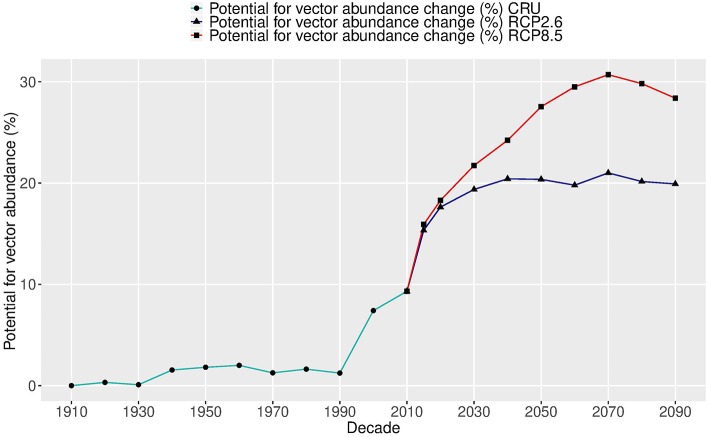
Relative changes (%) in the global potential abundance of *Aedes aegypti* over two centuries (1905–2099). The future change (starting in 2015) shows two carbon emission scenarios: RCP2.6 (blue lines) and RCP8.5 (red lines), using five global climate model ensembles (CMIP5) based on Model A.

The change in global average intensity of the potential abundance per decade was estimated over two centuries (1905–2099), as shown in Figure 4. During the past 110 years (1905–2014), the global abundance potential has increased about 9.5% as the global temperature has risen about 1.2°C. The largest increase (8.2%) occurred over the last two decades as a response to a rapid increase in temperature.

In the future under RCP2.6—the lowest carbon emission scenario—the potential vector abundance is projected to increase by at least the same amount as over the past 110 years, but during only a 20-year period, by 2030. After that, the potential of vector abundance levels off, following the temperature trend. The total vector abundance change over the two centuries is more than 20% and the temperature rise is about 2.6°C. The average increase per degree in this scenario is 8.2%.

Under the highest carbon emission scenario of RCP8.5, the change in the potential of vector abundance rises another 20% by the 2070s and then slightly decreases. The total change in vector abundance over the two centuries is more than 30%, and the temperature rise is about 5.8°C (1910–2070). The average increase per degree in this scenario is 5.0%. This is less than that under RCP2.6, due to saturation from temperatures that will be over the optimal value for vector development and survival. Therefore, two different future trajectories in the potential of vector abundance are observed under two carbon emission scenarios, with an average of 6.1% increase per degree. The potential abundance follows closely the temperature change for the same period with a correlation coefficient of 0.99 under RCP2.6 and 0.89 under RCP8.5.

### Sensitivity of Realized Abundance to Socioeconomic Development

**Figure 5:**
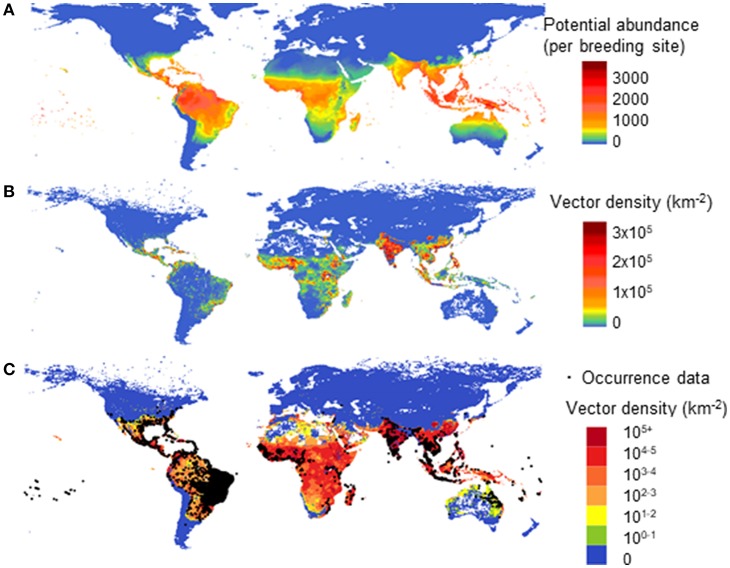
Model comparison of different drivers on the global distribution for potential abundance and vector density of female *Aedes aegypti*: using climate only (**A**—Model A) and climate, human population and GDPpc (**B,C**—Model B). **(A)** shows potential vector abundance (per larval site) averaged over the decade 2000–2009 in linear scale. **(B,C)** show population density averaged over 2006–2015 in linear scale **(B)** and in log scale where occurrence data was also overlaid **(C)**.

In a second model, using multiple drivers (Model B) we estimated the global distribution of vector population per area due to both climatic and socio-economic influences. Figure 5 shows a comparison of different driving forces for the global abundance of female adult *A. aegypti*, averaged over the past decade. In Figure 5A we use only climate as the driving force for vector abundance (per breeding site) using a linear scale. In Figure 5B (linear scale) and Figure 5C (log scale) (2006–2015) we use climate, human population, and GDPpc as the driving forces. Figure 5C overlays also the occurrence data (35).

In Figure 5B, similar to Figure 5A, the highest vector abundance is observed around the equator, in tropical areas. Figure 5B highlights the more densely populated vector areas with the linear color scale used, and thus emphasizes human influence on vector abundance in urban settings, such as Asia and Africa. The log scale in Figure 5C better illustrates the vector presence areas with low vector density, such as Australia, North Africa, and America. It compares better with the reported occurrence data, the black dots as shown in Figure 5C. The difference between Figures 5A,B is clearly shown in North Australia and the Amazon area of Brazil, where human population is low but the natural environment is suitable for this vector to flourish.

**Figure 6:**
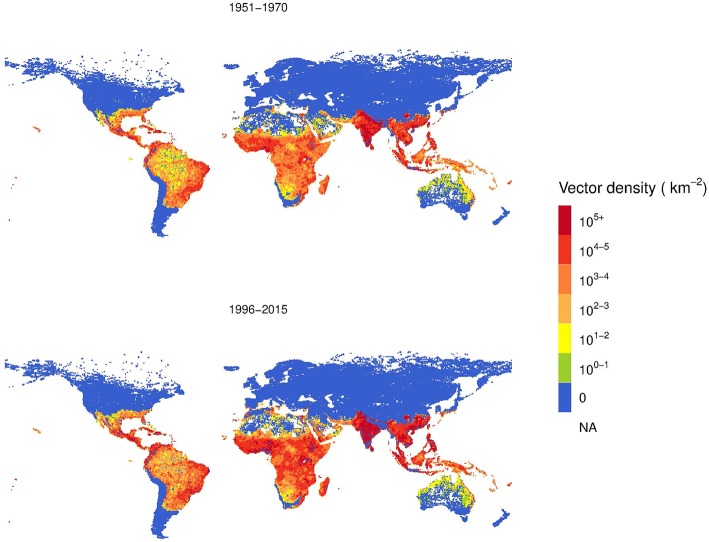
Comparison two-decades averaged global distribution of female *Aedes aegypti* vector density over 45 years using Model B.

Based on Model B, we estimated the global *A. aegypti* annual abundance averaged over two periods, 1951–1970 and 1996–2015, as shown in Figure 6. This analysis considers the change in climate, population and GDPpc. Over the 45-year time span, the vector density increased. The area with higher population density of *A. aegypti* is postdicted to spread from coastal areas toward more continental areas, following patterns of economic activity and higher human population density.

The log color scale is used to illustrate more clearly the low vector density areas, such as Australia and North America, where some increases are also observed.

## Discussion

This study uses a process-based mathematical vector model based on the lifecycle of *A. aegypti* to delineate the global distribution of vector potential abundance in space and time. We present two models: Model A postdicts and predicts potential abundance based on climate as the only driving force, and is built on an existing validated model structure (36) with improvements in model parametrization; Model B tests the sensitivity of the realized abundance of the vector to multiple driving forces consisting of both climate and socio-economic parameters, to account for human and economic influences on vector abundance. Using both models, we estimate vector population dynamics globally and present the change over the last and the current century.

Our study illustrates not only the global distribution of *A. aegypti*, but also the relative abundance, both geographically and temporally. Not surprisingly, seasons with the highest vector abundance are summer and fall, and the lowest are spring and winter. Over the past 110 years, the potential for vector abundance based on climate alone is estimated to have increased with about 9.5%. The largest increase (8.2%) has occurred since 1990, which indicates that a possible tipping point was reached in the global abundance of *A. aegypti*. In the future, as climate is projected to warm further, the potential for global distribution and abundance is also predicted to increase following the temperature trend closely, up to a 20% increase under a low emission future or a 30% increase under a high-emission future resulting in potential substantial changes in the abundance across the globe. Illustrating the effect of climate mitigation, the increase tapers off slowly after 2040 under the low carbon emission scenario, in agreement with the Paris Climate accord, and after 2070 under a high carbon emission scenario.

This is the first mathematical model to simulate the global abundance of *A. aegypti*. There are two distinct approaches to modeling global vector distribution: statistical models that compute the probability of presence/absence of vectors, and mathematical models, like the one presented here, that compute the vector population dynamics.

Our mathematical model tested the sensitivity of the climate-driven potential abundance to the realized global vector population distribution, based on human population and GDPpc. Information on human population was also included in the recent statistical model of global distribution of *A. aegypti* (17). We found the socio-economic sensitivity to the abundance to be large, which was also confirmed in the prior study, where it increased the probability of this vector's presence in areas of larger human population such as the coastal areas of the USA, eastern and southern Africa, and southern Asia.

Our study complements the statistical estimates based on presence/absence occurrence data collected from limited monitoring sources (14, 35). The most comprehensive available source for global distribution of *A. aegypti* is the recently published presence/absence occurrence data (35) for the period 1960–2014. All the models show generally similar regions but larger areas of vector presence than the vector surveillance data, especially in Africa and South Asia. This is reasonable, since the occurrence data can easily underestimate many areas of developing countries due to the underreporting of vector presence by deficient surveillance systems. There are considerable gaps in such vector surveillance. The eastern part of South America (e.g., Brazil) and the southern USA show more vector presence data, which may be due to a well-functioning surveillance system in response to frequent dengue or other epidemics, which our model captured. In addition, our model suggests more areas in Africa and Asia that, like Brazil, have even higher vector abundance but were not present in the occurrence data, possibly due to underreporting (35).

The statistical model estimates of global presence of *A. aegypti* from Kraemer et al. (14) are very similar to the result from our climate-driven model (see Figure S5A for comparison). Both used natural environmental factors, with climate as the dominant factor. However, in light of the dispersion of *Aedes* vectors to new areas through global trade (37, 38), and the creation of urban breeding sites owing to rapid urbanization and economic development (33, 39, 40), it is important to include human population and socio-economic factors when estimating the realized occurrence and abundance of the vector.

To the best of our knowledge, this study is the first mathematical model to study vector abundance on a global scale. Compared with climate-based models, this new model (Model B) should provide a complementary estimate of the distribution and a novel estimate of the abundance of *A. aegypti*.

Nonetheless, as with all modeling approaches, this study has limitations as well. Two such are the absence of capturing how irrigation, which is used to compensate for low rain levels, can exacerbate vector reproduction, and the use of air conditioning, which can lower vector survival and reproduction. These factors describing local variation are not included in this study on global vector population, although they are important to include for modeling a specific local area, as was done in some prior studies. For example, human-contributed semi-permanent water containers were included in the mathematical modeling for dengue transmission in San Juan, PR (41).

Another limitation is the lack of granularity of the climate data to sufficiently describe micro-climates, specifically urban heat islands (42), which may considerably increase the suitability of vectors in the fringe zones of vector abundance.

Although the original vector model has been validated in Campinas, Brazil against vector field data and dengue transmission data over a few decades (36), the vector field data is always limited and dengue transmission is indirect. Certain assumptions must be taken into account, especially for all the rainfall-dependent parameters, such as carrying capacity, hatching fraction, and mortality rates for underwater stages. In addition, without an egg stage, the fraction of eggs laid were assumed to transfer to the larval population instantaneously, which is unlikely. However, our time span for abundance estimation was at least 6 months after the introduction of the female adults. The transition of eggs to larvae has already occurred to a large extent, if breeding sites were available. According to an egg modeling study by Yang et al. (43), the eggs of *A. aegypti* have shown very complicated biological traits in hatching; indeed, eight compartments were used to model hatching alone. Therefore, it is doubtful that a four-stage model including eggs will enhance the results over the three-compartment model used here.

This study would have benefited more from vector population data to validate the model estimates, parameters, and to test for other possible relationships between model parameters, such as breeding site and climate/socio-economic variables. However, only very limited vector data from field measurement exist describing larval dynamics on a relative scale. Yang et al. set a fixed number of breeding sites of 1,000,000 for larva carrying capacity of *A. aegypti*. Other modeling studies of *A. albopictus* used 250,000 (21, 22). Due to a lack of vector population data to validate our model, except for limited vector presence areas, the values in vector abundance may be viewed as a relative measure of potential abundance driven by the factor incorporated in the models, rather than an absolute number.

Needless to say, vector abundance models are very sensitive to the parameters used in the models and the input climate dataset. Although model parameters were chosen carefully to validate a number of places where the vector were known to be present, caution must be taken when using these parameters for other datasets in the future. Finally, the equation for breeding sites may overestimate the human contribution and downplay the role of natural water bodies such as those in rainforests, in Model B. However, *A. aegypti* are anthropophilic mosquitoes and depend on humans for reproduction.

With all the limitations, the process-based mathematical model in this study has helped us to understand better the dependence of global *A. aegypti* distribution and abundance on the contribution of climate and socio-economic factors. This model may be used to disentangle other interactive effects among the climate factors and other factors (e.g., environmental) as well.

## Conclusion

Our study indicates high abundance of *A. aegypti* in many regions of the world with a tropical and subtropical climate. We suggest the highest abundance in South Asia, mid-Africa and South America, and a clear seasonal pattern. Incorporating socio-economic factors, the global distribution and abundance show pronounced clustering of the vectors around densely populated urban areas, reflected by the abundance of potential food (blood meals) and human-created larval sites. Over the last century, *A. aegypti* is postdicted to have expanded the suitable areas for its habituation and abundance. The increase in the abundance of this vector correlates closely with the global temperature increase. The change in the potential of global abundance over the last century up to today is estimated to be an increase of 9.5% globally and a further increase to 20 or 30% under a low compared to a high carbon emission future, respectively. The largest increase to date has occurred in the last two decades, indicating a tipping point in climate-driven global abundance which is stabilized at the earliest in the mid-tweny-first century. Our findings indicate that climate change mitigation would considerably suppress vector abundance in the second half of the twenty-first century. Following the Paris Agreement has a large impact on the potential abundance of this potent vector, *A. aegypti*, and therefore may prevent many severe infectious diseases epidemics in the near future. Our study further indicates that the actual abundance and distribution are sensitive to socio-economic development.

## Data Availability

Publicly available datasets were analyzed in this study. This data can be found here: ISIMIP (https://esg.pik-potsdam.de/projects/isimip/), CRU (http://www.cru.uea.ac.uk/data/).

## Author Contributions

JL-H carried out the model development and the implementation and drafted the manuscript. MS downloaded climate and human population data, translated the Mathematica code to R, and generated the maps and figures. JR conceived the research and helped with modeling and drafting of the manuscript. ÅB guided the modeling and helped with the analysis of the model. JS helped with the writing of the paper. All authors discussed the results and contributed to the revision of the final manuscript.

### Conflict of Interest Statement

The authors declare that the research was conducted in the absence of any commercial or financial relationships that could be construed as a potential conflict of interest.
