# Supplementary material for: Estimating Past, Present, and Future Trends in the Global Distribution and Abundance of the Arbovirus Vector Aedes aegypti Under Climate Change Scenarios
Source: Front Public Health. 2019 Jun 12;7:148. doi: 10.3389/fpubh.2019.00148 (PMC6582658; doi:10.3389/fpubh.2019.00148)
Supplement: Supplementary file 1 [file Presentation_1.pdf]

Supplementary Material

1 Modelling framework

We used a process-based mathematical model with three compartments as shown in Figure S1 based on the lifecycle of *Aedes aegypti*: Larvae, Pupae, and Adults (1). Two models variants are used in this study: Model A uses only climate as the driving force (Figure S1A); Model B uses climate, GDP and human population as the driving forces (Figure S1B).

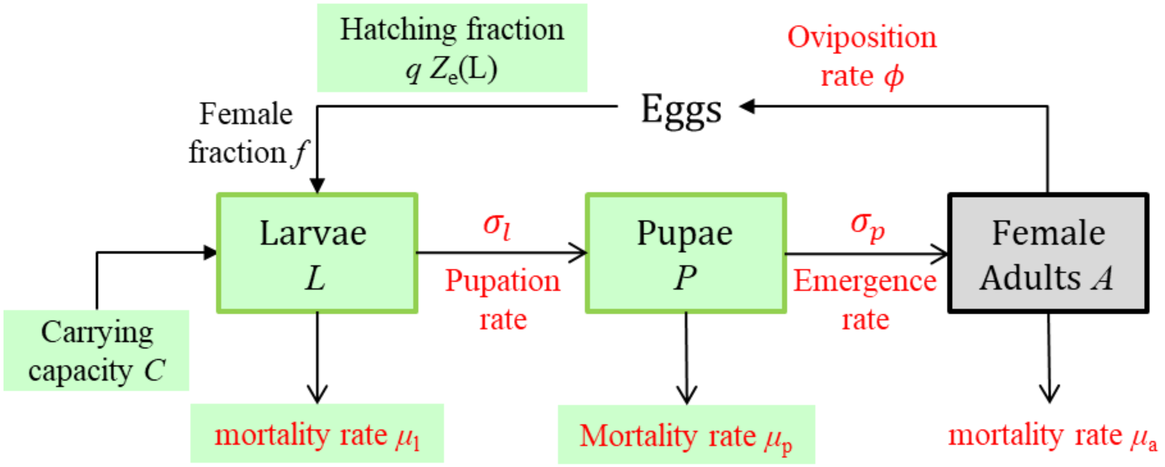

Figure S1A. Model A framework for *Aedes aegypti* mosquito population dynamics. Colour codes are: shaded green is precipitation dependent or under-water stages, red coloured words temperature dependent. Three vector populations are in boxes. Model parameters are those boxes without borders, which describe all the vital rates, and environmental influences through the carrying capcity.

All the mosquitoes' vital rates (birth, death, and transition) depend on temperature and/or precipitation. In addition, the egg-to-larva hatching fraction depends on the larvae population and environmental carrying capacity due to competition for survival. The whole lifecycle from egg to adult takes one to a few weeks on average depending on temperature and precipitation.

Three ordinary differential equations are used to describe the rates of change of individuals for each of the life-history stages:

$$\frac{\mathrm{d}L}{\mathrm{d}t} = \Phi A q Z_e(L) f - \mu_l L - \sigma_l L, \qquad (1A)$$

$$\frac{\mathrm{d}P}{\mathrm{d}t} = \sigma_l L - \mu_p P - \sigma_p P, \qquad (2)$$

$$\frac{\mathrm{d}A}{\mathrm{d}t} = \sigma_p P - \mu_a A. \qquad (3)$$

Here *L*, *P*, and *A* are the total female larval, pupal, and adult population per area, with the area being each 0.5 x 0.5 degree grid from which we generate our global maps. All the variables and parameters used in these equations are explained in Table S1-3.

From the top down, the three equations describe the rates of change of their respective densities. Female adults lay eggs at rate Φ of which a fraction $qZ_e(L)$ are viable, of which a fraction f become female. Larvae die at rate μ_l and develop into pupae at rate σ_l. Similarly, pupae die at rate μ_p and emerge as female adults at rate σ_p. Adults die at rate μ_a.

The egg-to-larva hatching fraction is expressed as the product of two factors: the fraction of eggs hatching q at low larval population density and $Z_e(L) = 1 - L/C$ the larval density-dependent reduction factor for the viable eggs to survive and develop into larvae if larval population density is under the environmental carrying capacity C. In principle, L can be greater than C in which case we consider the hatching rate to be zero, but it did not happen in any of the cases we consider.

Model B includes human contributions to both the larval breeding sites and blood meals for the mosquito's reproduction, the fecundity rate of adult females as the shaded yellow colour (Figure S1B). GDP is also included in contributing to the mosquito's larval sites.

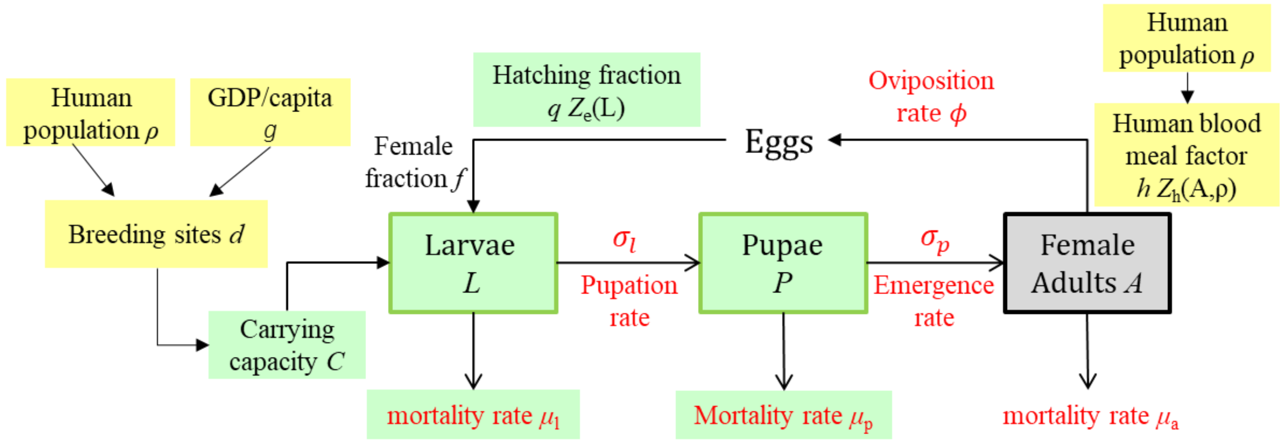

Figure S1B. Model B framework for *Aedes aegypti* mosquito population dynamics. Shaded yellow indicates human population or activity related parameters. Three vector populations are in boxes. Additional model parameters (shaded yellow) are used to describe the environmental and human influences.

In Model B, only Equation (1A) is changed to account for human population and gross-domestic product (GDP) per capita:

$$\frac{\mathrm{d}L}{\mathrm{d}t} = \Phi hAqZ_e(L)f - \mu_l L - \sigma_l L. \qquad (1B)$$

Here, one additional factor is added to the first term to describe the eggs laid by the female adults at rate Φh. Larvae die at rate μ_l and develop into pupae at rate σ_l. Similarly, pupae die at rate μ_p and emerge as female adults at rate σ_p. Adults die at rate μ_a.

The adult female fecundity rate Φh is expressed as the product of two factors. The first is the oviposition rate Φ as determined in laboratory studies when sufficient blood meals were provided. Under natural conditions, the availability of blood meals is limited by human population density ρ, as

described by the second factor *h* (human blood-meal factor), which describes the reduction in oviposition rate due to lack of available blood meals.

The colour codes are similar to the framework in Figure S1, with red representing temperature-dependent variables, green rainfall-dependent variables, yellow human- dependent parameters or variables, grey vector population density, and white constant or mixed variable types. We used a pseudo-unit [M] for mosquito numbers (2) with all other units conforming to the SI system.

2 Variables and their relations with weather, human population and GDP

2.1 Parameterization

The variables and functions used in Equations (1-3) are summarized as equations (4-11), see Table S1A for variables used in Model A and Table S1B for Model B. The parameters and values used in these equations are summarized in Table S2 and S3. Each function is motivated and explained here.

The human blood meal factor *h*, Equation (4) is used based on the fact that in nature, except for rainforests, humans are the main food that *Aedes aegypti* require for laying eggs (3). ρ_1 corresponds to the human population density at which half blood meal is obtained. The value was taken as the minimum human density within the flying distance of *Aedes aegypti*, one person per 50 x 50 meter (Table S1B) (2). Equation (6) is the fraction of eggs that hatch to larvae, *q*, in the situation where there are sufficient larval sites for hatching. This under-water process takes place from both the rainfall-dependent larval site (first term) and the rainfall-independent larval sites (q_2 – see Table S2) (2).

Larval population is regulated by the environmental carrying capacity (*C*). *C* is expressed as the product of two factors, *C*=*cd*. Here, *c* as shown in Equation (7) is the carrying capacity per larval site and is mainly contributed by rainfall (*W*) with a small fraction from sources independent of rainfall (c_2) (2). *d* as shown in Equation (5) is the number of larval sites that comes from human contribution, directly or indirectly through agriculture, polyculture and urbanization(4). Directly, humans create the untreated water containers that catch rain water, i.e., for water storages used for drinking, farming, and husbandry, or used tires and waste containers (4, 5). In general, we assume that the higher the human population in a given area (ρ), the more the larval sites (*d*). The lower the economic level, the more the water containers for larval site. We assume that *d* is proportional to the human population and inversely proportional to the economic level, which we approximate by the square root of the ratio of GDP/capita (g) to the world average value (g_a). In Equation (5), the proportional coefficient, *b*, is used to represent the number of larval sites per person if the GDP/capita is at the world average value. *b* is determined by comparing the model output with the field data for Juaweiro, Brazil – see Table S2.

Equations (9-10) describe the mortality rates of larva and pupa. They depend on both temperature and precipitation since they are under-water stages. Heavy rainfall washes out the larval site through overflow and creates extra mortality if it exceeds the critical value W_c – 30 mm/day (2). In the equations, $\theta(x)$ is the Heaviside function which is 1 if $x \geq 0$ and 0 otherwise. The temperature dependent part of the mortality $\mu(T)$ as well as the development/transition rate (σ) between compartments and the intrinsic oviposition rate ($\boldsymbol{\Phi}$) are estimated from laboratory studies of *Aedes aegypti* (6). They are fitted using *n*-th degree polynomial as shown in Equation (8) with coefficients shown in Table S3, where y(*T*) stands for either $\mu_j(T)$, $\sigma_j(T)$ or $\boldsymbol{\Phi}(T)$, and *j* stands for either l, p, or a, denoting the three stages of the mosquito's life.

Table S1A. Summary of symbols used in Model A, their meanings, units, values and sources.

Symbol	Meaning	Unit	Value or Expression	Equation number	Source
f	fraction of eggs that can become female mosquitoes		0.5		
$Z_e(L)$	density-dependent reduction factor for egg-larva hatching		$1-L/C$	11	
t	time	day			
$L(t)$	female larval population	[M]/breeding site		1A	
$P(t)$	female pupal population	[M]/breeding site		2	
$A(t)$	female adult mosquito population	[M]/breeding site		3	
$W(t)$	daily precipitation	mm/day	time series		data input [i, ii]
$q(W)$	fraction of eggs hatching to larvae		$q(W) = \frac{q_1 W}{q_0 + q_1 W} + q_2$	6	Eq.(2) in Ref. (2)*
$c(W)$	carrying capacity for larvae per breeding site	[M]/breeding site	$c(W) = \frac{c_2 W}{c_1 + W} + c_2$	7	Eq.(2) in Ref. (2)*
$C(W)$	environmental carrying capacity for larvae	[M]/breeding site	$C=c$		
$T(t)$	mean daily temperature	°C	time series		data input [i, ii]
$\boldsymbol{\Phi}$(T)	intrinsic oviposition rate per female mosquito	day^{-1}	$y(T) = \sum_{i=0}^{n} p_i T^i$	8	Table 3.2 in Ref. (6)**
$\sigma_l(T)$	per-capita transition rate from larva to pupa	day^{-1}	$y(T) = \sum_{i=0}^{n} p_i T^i$	8	Table 3.2 in Ref. (6)**
$\sigma_p(T)$	per-capita transition rate from pupa to adult	day^{-1}	$y(T) = \sum_{i=0}^{n} p_i T^i$	8	Table 3.2 in Ref. (6)**
$\mu_a(T)$	per-capita mortality rate of female adults	day^{-1}	$y(T) = \sum_{i=0}^{n} p_i T^i$	8	Table 3.2 in Ref. (6)**
$\mu_l(T,W)$	per-capita mortality rate of larvae	day^{-1}	$\mu_l(T, W) = \mu_l(T)\{\theta(W - W_c)\, \mu_{al}(W - W_c) + 1\}$	9	Eq.(3.2) in Ref. (6)**
$\mu_p(T,W)$	per-capita mortality rate of pupae	day^{-1}	$\mu_p(T, W) = \mu_p(T)\{1 + \mu_{pa}[W - W_c]\, \theta[W - W_c]\}$	10	Eq.(3.2) in Ref. (6)**

[i] ISMIP gridded annual (ρ, g) and monthly (T, W) data during 1950-2099.
[ii] CRU TS3.25 gridded monthly data during 1901-2015.
* Based on relations from Yang et al. (2016) (2).
** Fitted to data in laboratory studies from Yang et al. (2014 Chapter 3) (6).
Values of parameters for which no source is given are discussed in this paper.

Table S1B. Summary of symbols used in Model B, their meanings, units, values and sources.

Symbol	Meaning	Unit	Value or Expression	Equation number	Source
f	fraction of eggs that can become female mosquitoes		0.5		
$Z_e(L)$	density-dependent reduction factor for egg-larva hatching		$1-L/C$	11	
t	time	day			
$L(t)$	female larval population	$[M]/km^2$		1B	
$P(t)$	female pupal population	$[M]/km^2$		2	
$A(t)$	female adult mosquito population	$[M]/km^2$		3	
$\rho(t)$	human population density	$persons/km^2$	time series		data input [i]
ρ_1	half-saturation constant	$persons/km^2$	100		constant
$g(t)$	GDP/capita	$/person	time series		data input [i]
$g_a(t)$	world average GDP/capita	$/person	time series		data input [i]
$h(\rho)$	human blood meal factor		$h(\rho) = \frac{\rho}{\rho + \rho_1}$	4	
$d(\rho, g)$	larval sites related to human activities	$breeding\ site/km^2$	$d(\rho, g) = b\rho \sqrt{\frac{g_a}{g}}$	5	
$W(t)$	daily precipitation	mm/day	time series		data input [i, ii]
$q(W)$	fraction of eggs hatching to larvae		$q(W) = \frac{q_1\ W}{q_0 + q_1\ W} + q_2$	6	Eq.(2) in Ref. (2)*
$c(W)$	larval carrying capacity per breeding site	[M]/breeding site	$c(W) = \frac{c_2\ W}{c_1 + W} + c_2$	7A	Eq.(2) in Ref. (2)*
$C(W, \rho, g)$	environmental carrying capacity for larvae	$[M]/km^2$	$C(W,\rho,g) = c(W)d(\rho,g)$	7B	
$T(t)$	mean daily temperature	°C	time series		data input [i, ii]
$\boldsymbol{\Phi}(T)$	intrinsic oviposition rate per female mosquito	day^{-1}	$y(T) = \sum_{i=0}^{n} p_i\ T^i$	8	Table 3.2 in Ref. (6)**
$\sigma_l(T)$	per-capita transition rate from larva to pupa	day^{-1}	$y(T) = \sum_{i=0}^{n} p_i\ T^i$	8	Table 3.2 in Ref. (6)**
$\sigma_p(T)$	per-capita transition rate from pupa to adult	day^{-1}	$y(T) = \sum_{i=0}^{n} p_i\ T^i$	8	Table 3.2 in Ref. (6)**
$\mu_a(T)$	per-capita mortality rate of female adults	day^{-1}	$y(T) = \sum_{i=0}^{n} p_i\ T^i$	8	Table 3.2 in Ref. (6)**
$\mu_l(T,W)$	per-capita mortality rate of larvae	day^{-1}	$\mu_l(T, W) = \mu_l(T)\ \{\theta(W - W_c)\ \mu_{al}(W - W_c) + 1\}$	9	Eq.(3.2) in Ref. (6)**
$\mu_p(T,W)$	per-capita mortality rate of pupae	day^{-1}	$\mu_p\ (T, W) = \mu_p\ (T)\ \{1 + \mu_{pa}[W - W_c]\ \theta[W - W_c]\}$	10	Eq.(3.2) in Ref. (6)**

[i] ISMIP gridded annual (ρ, g) and monthly (T, W) data during 1950-2099.
[ii] CRU TS3.25 gridded monthly data during 1901-2015.
* Based on relations from Yang et al. (2016) (2).
** Fitted to data in laboratory studies from Yang et al. (2014 Chapter 3) (6).
Values of parameters for which no source is given are discussed in this paper.

Table S2. Precipitation-dependent model parameters, values, and sources.

Symbol	Meaning	Unit	Value	Source
c_2	rain-independent larval sites	[M]/larval site	0.1	Table 2 in Ref. (2)*
c_0	production of breeding sites by rain	[M]/larval site	5	Table 2 in Ref. (2)*
c_1	amount of rain to produce 1/2 of the max breeding sites	mm/day	30	Table 2 in Ref. (2)*
q_0	amount of rain to allow 50% of eggs hatching	mm/day	0.2	Table 2 in Ref. (2)*
q_1	capacity of eggs hatching with rain		0.02	Table 2 in Ref. (2)*
q_2	hatching fraction from rain-independent sites		0.037	
μ_{la}	additional mortality for larvae due to heavy rain	day/mm	0.001	**
μ_{pa}	additional mortality for pupae due to heavy rain	day/mm	0.001	**
W_c	critical rain volume to cause additional mortality	mm/day	30	Table 2 in Ref. (2)*
b	the number of larval sites per person if GDP/capita=ga		2.744	Calibration to field data***

* Based on study by Yang et al. (2016) (2).
** Modified from the values used by Yang et al. (2016) (2).
*** Modelled in this study but calibrated against field data in Juaweiro, Brazil (2015) (7).

Table S3. Coefficients (p_i) in temperature-dependent vector parameters obtained by fitting polynomial (2). The unit of coefficients p_i is days^{-1} × (°C)$^{-i}$.

Coefficient	Φ	μ_l	μ_p	μ_a	σ_l	σ_p
p_0	-5.40	2.32	4.25×10^{-1}	8.69×10^{-1}	-1.84	21.90
p_1	1.80	-4.19×10^{-1}	-3.25×10^{-2}	-1.59×10^{-1}	8.29×10^{-1}	-10.31
p_2	-2.12×10^{-1}	2.73×10^{-2}	7.06×10^{-4}	1.12×10^{-2}	-1.46×10^{-1}	2.05
p_3	1.02×10^{-2}	-7.53×10^{-4}	4.39×10^{-7}	-3.41×10^{-4}	1.31×10^{-2}	-2.24×10^{-1}
p_4	1.52×10^{-4}	7.50×10^{-6}		3.81×10^{-6}	-6.46×10^{-4}	1.47×10^{-2}
p_5					1.79×10^{-5}	-5.89×10^{-4}
p_6					-2.62×10^{-7}	1.41×10^{-5}
p_7					1.5×10^{-9}	-1.85×10^{-7}
p_8					-	1.0×10^{-9}

2.2 Implementation

Figure S2 illustrates the method used in this study as a flow diagram from data input to model output. Results are presented as global maps and trend. The top row shows the process. Here the vector model was developed in two steps: Model A, climate driving the vector population (Fig 6A); Model B, climate, human population and GDP driving together (Fig 6B).

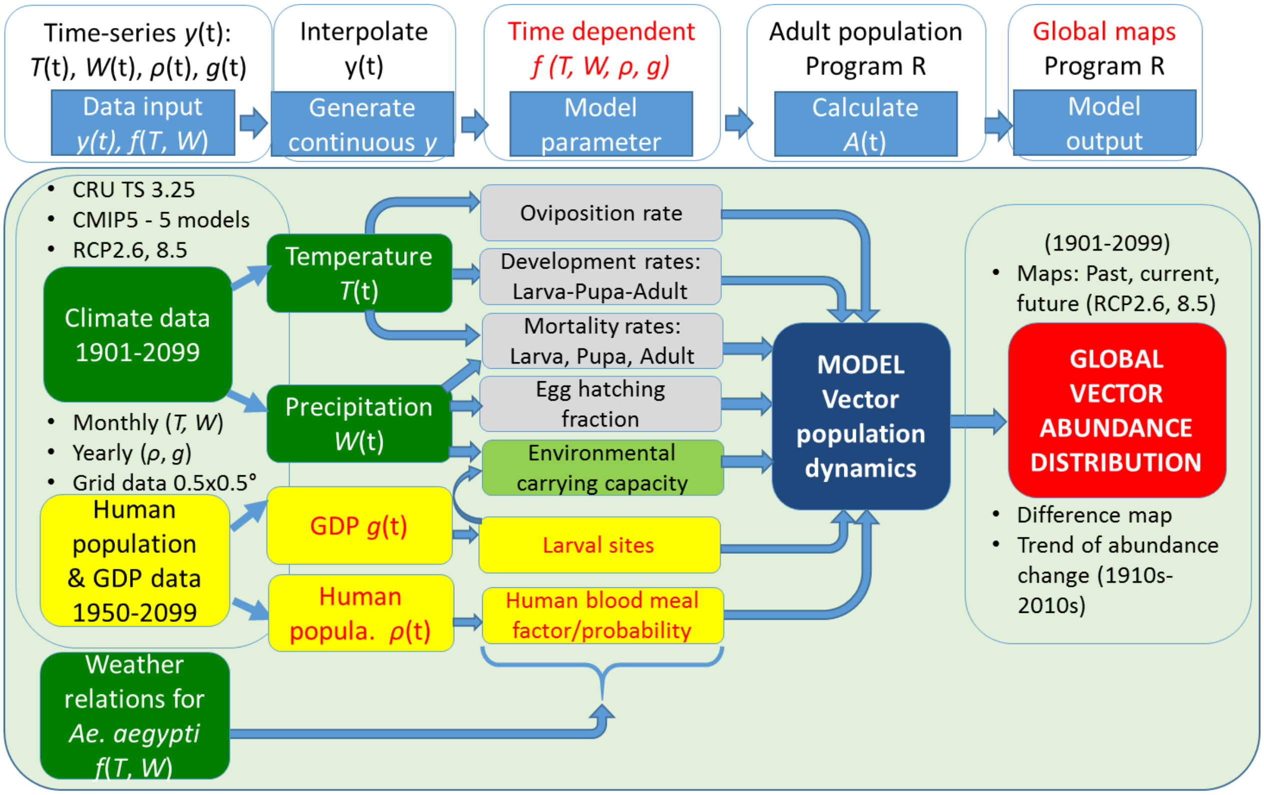

Figure S2. The method flow diagram: from data input to model output. The top row shows the process. Climate, human population and GDP data sets are interpolated first to obtain 4-continuous variables before linking to the vector development related parameters. These parameters are then used as inputs to the model to calculate vector population dynamics. Average female adult mosquito population over a certain period is defined as the vector abundance. From this, a global map of vector abundance distribution is generated.

In Model A, only climate data are used as the input with two variables: mean temperature and precipitation. These two monthly datasets are then interpolated to generate continuous climate data. Weather relations of eight model parameters are obtained from the literature (Equations (6-7, 8-10)) with some improvement to be consistent with the assumptions made. Next, the climate data are linked to these model parameters before entering into the model. Six of the eight model parameters depend on temperature – see Figure S1. Precipitation (rainfall) contributes to the under-water stage of vector development, from mortality rate, egg-hatching fraction to environmental carrying capacity. Here the environmental carrying capacity for larvae is affected by the larval sites created by both rainfall, nature, and human activities (see Table S1B).

In Model B, human population and GDP are two extra datasets used as input into the model. They describe the socioeconomic factor that contributes to the vector's development through two model parameters (yellow boxes). First, they both contribute to the number of larval sites, assuming a direct proportion of the number of larval sites to human population and an inverse proportion to GDP per capita (Equation (5)). Second, human population provides blood meals to the urban vector; this is described as the human blood meal factor (Equation (4)). Like the climate data, the socioeconomic datasets are interpolated and linked to these two model parameters before being entered into the Model.

From the Climate Research Unit (CRU) online database, time series (CRU TS3.25) of gridded (0.5 x 0.5 degrees) monthly mean temperature and precipitation were downloaded from January 1, 1901 to December 31, 2016 (8). From ISMIP (9), we downloaded the future climate data from five global circulation models (CMIP5) (10) for RCP2.6 and RCP8.5 (2006-2099), the yearly global human population (1901-2099) and GDP (1950-2099) data. Both are gridded data at 0.5° and 5' resolutions. We use linear interpolation function to obtain continuous data before solving the equations.

Programs *Wolfram Mathematica* 11 and *R* (package deSolve 1.20) (11) are used to obtain time series vector population and abundance values and to generate global maps.

To obtain the vector populations, we solve differential equations (Eq. (1-3)) numerically using the two computer programs mentioned above. From this, we summarize the total female adult vector population over a defined period to get vector abundance potential from Model A output or abundance density from Model B output. Finally, based on averaged seasonal or annual vector abundance over a decade, we map the vector abundance globally. The results from the two models A and B are compared to see the additional contribution to vector abundance from socioeconomic factors in addition to climate. In addition, we regressed the percentage change in global vector abundance potential against changes in global mean temperatures per decade over two centuries (1901-2099) using linear regression. The future two climate scenarios were used, RCP 2.6 and RCP 8.5. The resulting regression coefficient gave the average percentage change in vector abundance potential in relation to unit increase in temperature while the R^2 provided the correlation coefficient.

3 Optimization of model parameters

Two new model parameters were used in Model B to generate vector population density besides those used for Model A: b - the number of larval sites for larva at a given human population density if GDP/capita is the same as the world average and ρ1 – the half-saturation constant to allow gravid female mosquitos to feed human blood for laying eggs. Based on the published field study on Aedes aegypti population (7), we have calibrated these two parameters by comparing the model output of female adult with the field data on the same capture days (Table S2). See Figure S3 for the comparison of field data and our model 2 output.

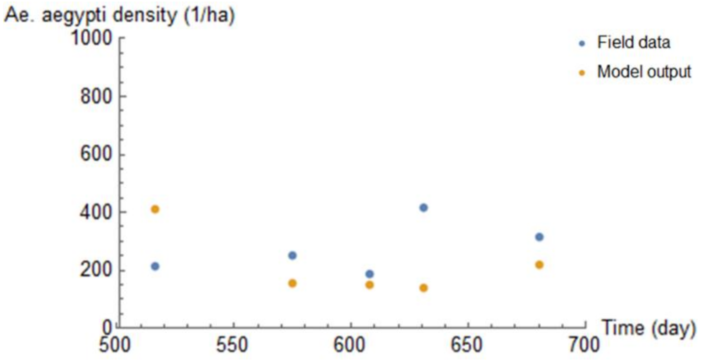

Figure S3. Comparison of female adult *Aedes aegypti* density from model output to the field data on the five different days of the year 2011. The initial day for the introduction of one female mosquito was 2010.1.1 and model output on the capturing days were shown at days 516 (31/05), 575 (29/07), 608 (31/08), 631 (23/09), and 680 (11/11) in 2011. Additional data used in the model were local human population (165/ha) from the study area (7) and GDP/capita for Bahia state where Juaweiro is located ($11340.26) (12) and for the world ($10151) (13) in 2011.

4 Sensitivity analysis of model parameters

We have used nine time-varying parameters in the model B. Besides the two parameters related to human population and socioeconomics as described above, the rest seven parameters were climate related. They are listed in Table S1B as Equations 4, 6, 7B, 8-10. Some of these parameters were measured in laboratories (6) and some were assumed and validated to local dengue outbreak cases (1, 2). Here we perform sensitivity analysis to see the effect of each parameter to the output of Model B – the abundance of the adult vector population annual average over the recent 10 years for six locations.

Each parameter is varied 1000 times sampling around its original value following a normal distribution with sigma of 0.05. We used R-package (pse v.0.4.7) to run the model (14). The results were generated as empirical cumulative density functions and partial rank correlation coefficients (PRCC) between a set of model input parameters and the output of *Aedes aegypti* adult abundance. Figure S4 shows PRCC for six cities across different climate and locations. The top panel shows three areas with *Aedes aegypti* endemicity; the low panel corresponds to relatively mild climate areas where *Aedes aegypti* has been found in Madeira since 2005 (15) and Phoenix recently (16).

The result shows that adult mortality rate, μ_m, is consistently the most sensitive parameters in affecting the abundance of adult population. Environmental carrying capacity, *C*, for larvae and the development rate from larva to pupa, σ_l, are two important parameters for all areas except Madeira and Malaga where vector population is low due to low temperatures when using CRU TS3.25 dataset without coastal temperature corrections applied. In addition, three more parameters are sensitive for the low panel but not the upper one: the oviposition rate θ_m, the egg hatching fraction *q*, and *h*, the human blood meal factor.

Therefore, we have found that sensitivity of parameters to the adult abundance depends on the level of vector abundance. The high abundance areas is sensitive to the limiting factor from environment carrying capacity, while low abundance areas is sensitive to the oviposition rate and hatching fraction. Overall, the adult vector mortality rate is the sensitive parameter to the adult population, as expected.

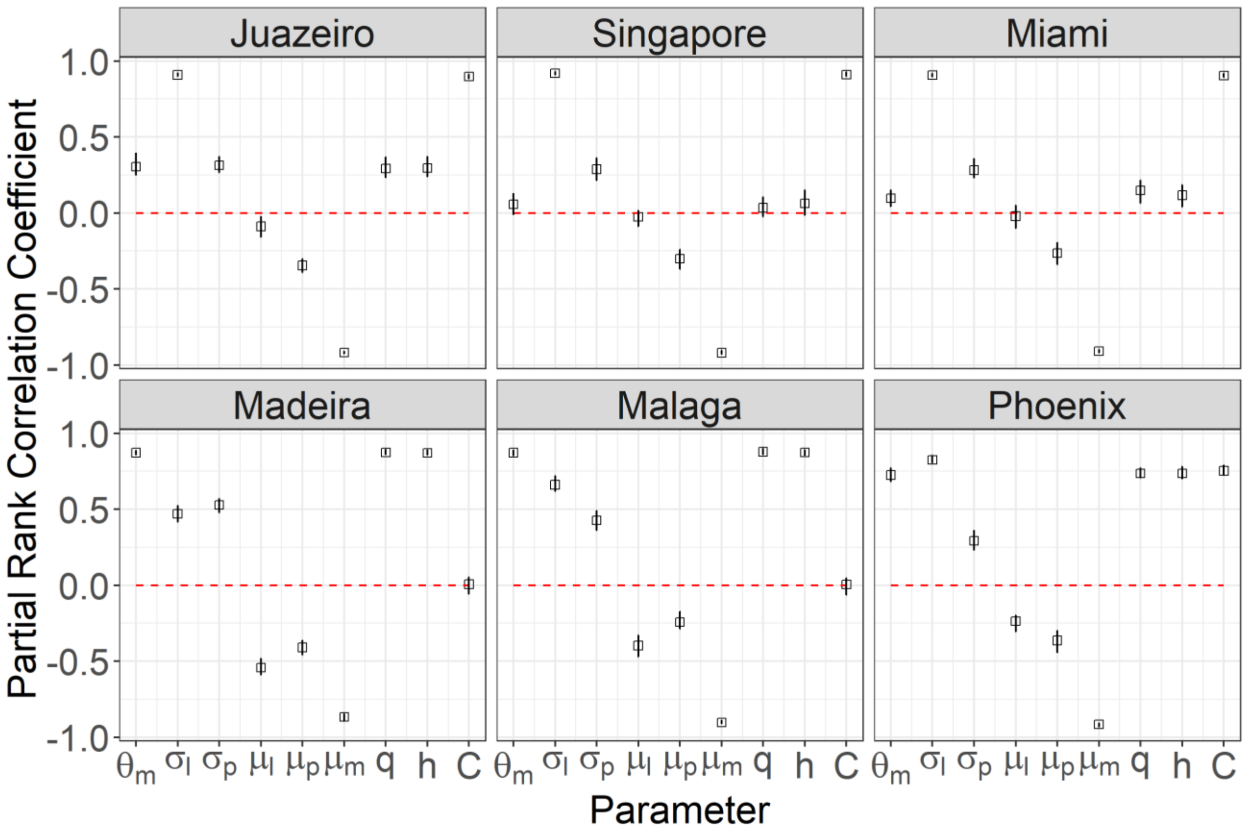

Figure S4. Sensitivity analysis using Partial Rank Correlation Coefficient (PRCC) over six cities in the world (17). The output variable is the annually averaged female adult abundance of *Aedes aegypti* over the recent decade 2006-2015. Vector was introduced in 2001.6.1. The nine model parameters are: θ_m -oviposition rate, σ_l - development rate from larva to pupa, σ_p - development rate from pupa to adult, μ_l, μ_p, μ_m – larval, pupal and adult mosquito mortality rate, q – eggs hatching fraction, C - environmental carrying capacity and h – the human blood meal factor.

5 Validation – comparing global map of invasion vs. occurrence data

Validation of our model has been carried out through comparing the model output - *Aedes aegypti* decadal abundance predictions - to the reported data on global occurrence of *Aedes aegypti* (18). As shown in Figure S5, black dots are the occurrence data. Different colors represent different values from the model output of population abundance potential of female adult vector per breeding site (Fig. S5A from Model A in linear scale) and of female adult vector population density (Fig. S5B from Model B in log scale) (2006-2015): blue corresponds to zero population density or abundance potential; green, yellow, orange and red correspond to positive density or abundance potential with increasing values.

To a large degree, we find a good overlap between our *Aedes aegypti* abundance predictions and empirical observations of its occurrence in Asia, America and Australia. The overlap is better for the vector density output from Model B than the abundance potential from Model A. The discrepancy is more in Africa and southeast Asia, as well as Northwest of Australia. Our density model output matches Brazil very well but not in Africa and Southeast Asia. This is probably due to incomplete monitoring and data collection system for the *Aedes aegypti* data. The monitoring system is much better in Brazil

than Asia and Africa. Therefore, we believe that our prediction is reasonable. In Australia, the discrepancy can be due to other reasons, such as lacking introduction of vectors and their spontaneous die out. Those factors are not included in our deterministic mechanical models.

The differences between Fig. S5A and S5B indicates the human contribution to the *Aedes aegypti* population growth. Fig. S5A is from Model A which is climate driven. Fig. S5A showing the vector population potential captures largely the occurrence data globally except some small areas such as part of Australia and middle of USA. This means that vector population development is largely controlled by climate.

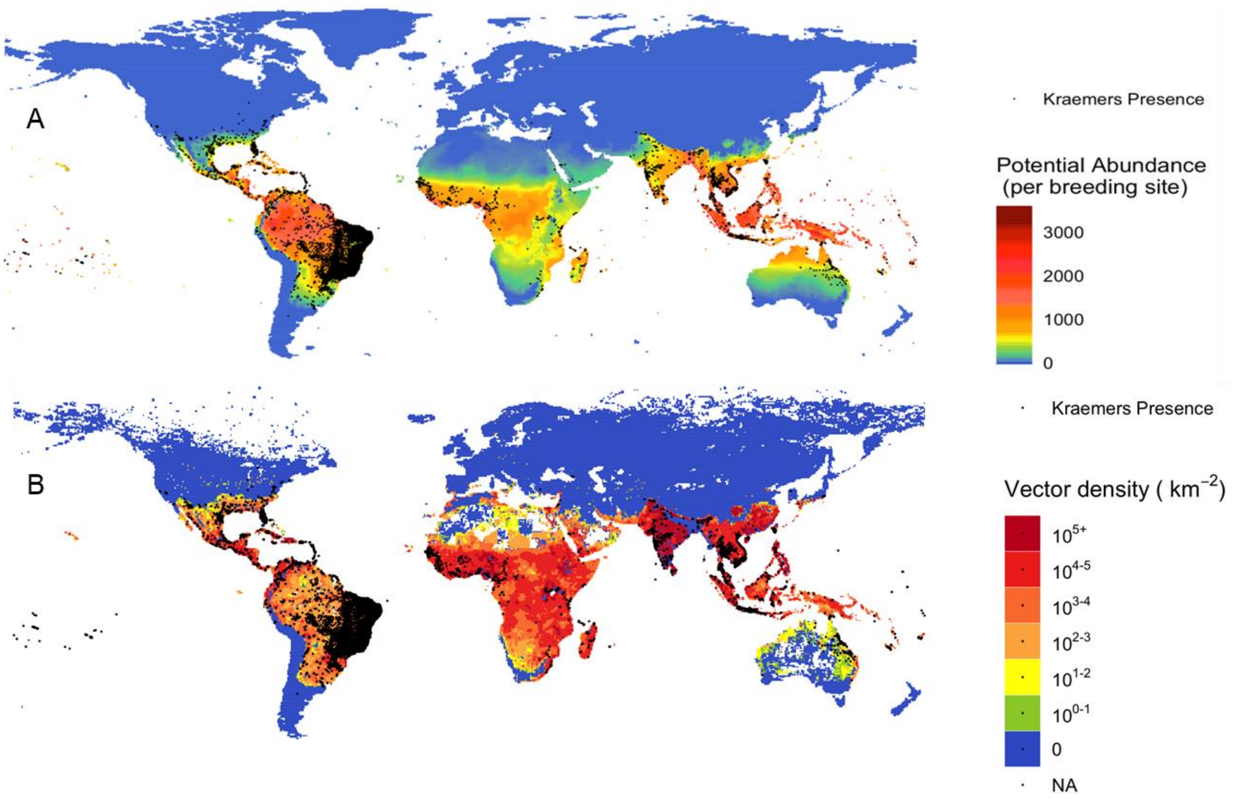

Figure S5. Comparison of model outputs on global map of *Aedes aegypti* abundance potential (A) and density (B) (continuous colors) and occurrence data (black dots) (18). Different drivers were used in the models: using climate only (Fig. S5A - Model A) and climate, human population and GDPpc (Fig. B – Model B). Parameter inputs were based on interpolated CRU TS3.25 monthly mean temperature and precipitation data. Female *Aedes aegypti* population potential abundance per larval site (Figure S5A, linear scale) and density (Figure S5B, log scale) were averaged over the period 2006-2015.

Figure S6 shows the percentage of observed occurrence areas at each category of potential abundance predicted by the model A. We found that within the occurrence data area, 97.5% of the global occurrences are within areas with potential abundance predicted above 300 and about 55% of occurrence data lies within abundance potential between 900-1200. Therefore, our model A predicted potential abundance agrees well with global observations of vector occurrence data.

The occurrence data need to be interpreted with caution as they are not systematically sampled. Thus, more data points in a specific region is likely to reflect the frequency of measuring and reporting rather

than the actual occurrence. See Kraemer et al for more information and discussion on data collection and validity (18).

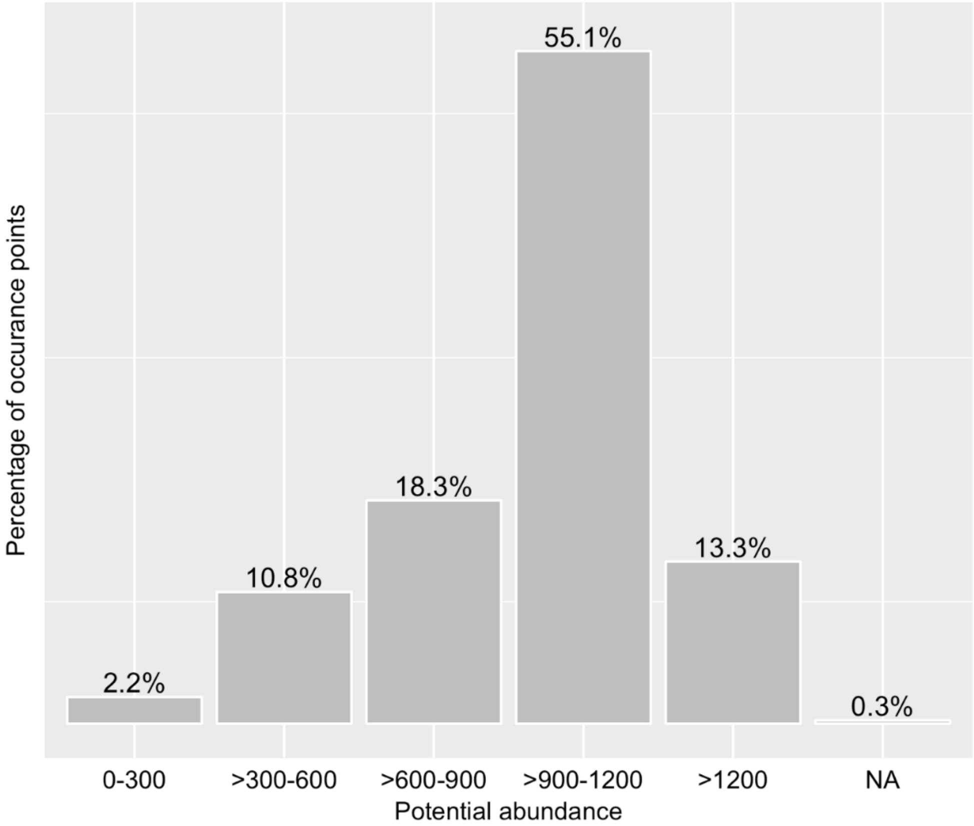

Figure S6. Distribution of the occurrence data[18] observations by categories of global potential abundance. Parameter inputs were based on Interpolated CRU TS3.25 monthly mean temperature and precipitation data and potential abundance was averaged for the period 2006-2015.

References

1. Yang HM, Boldrini JL, Fassoni AC, de Lima KKB, Freitas LFS, Gomez MC, et al. Abiotic effects on population dynamics of mosquitoes and their influence on dengue transmission. Ecological Modelling Applied to Entomology: Springer; 2014. p. 39-79.
2. Yang HM, Boldrini JL, Fassoni AC, Freitas LFS, Gomez MC, de Lima KKB, et al. Fitting the incidence data from the City of Campinas, Brazil, Based on Dengue Transmission Modellings Considering Time-Dependent Entomological Parameters. PloS one. 2016;11(3):e0152186.
3. Siriyasatien P, Pengsakul T, Kittichai V, Phumee A, Kaewsaitiam S, Thavara U, et al. Identification of blood meal of field caught *Aedes aegypti* (L.) by multiplex PCR. Southeast Asian J Trop Med Public Health. 2010;41(1):43-7.
4. Zahouli JBZ, Utzinger J, Adja MA, Müller P, Malone D, Tano Y, et al. Oviposition ecology and species composition of Aedes spp. and *Aedes aegypti* dynamics in variously urbanized settings in arbovirus foci in southeastern Côte d'Ivoire. Parasites & Vectors. 2016;9(1):523.
5. Reiter P. Aedes albopictus and the world trade in used tires, 1988-1995: the shape of things to come? J Am Mosq Control Assoc. 1998;14(1):83-94.
6. Yang HM, Macoris ML, Galvani KC, Andrighetti MT, Wanderley DM. Assessing the effects of temperature on the population of Aedes aegypti, the vector of dengue. Epidemiol Infect. 2009;137(8):1188-202.
7. Carvalho DO, McKemey AR, Garziera L, Lacroix R, Donnelly CA, Alphey L, et al. Suppression of a field population of Aedes aegypti in Brazil by sustained release of transgenic male mosquitoes. PLoS neglected tropical diseases. 2015;9(7):e0003864.
8. Harris IC, Jones PD, Osborn TJ, Lister DH. Updated high-resolution grids of monthly climatic observations – the CRU TS3.10 Dataset. Int J Climatol. 2014;34(3):623–42.
9. Input data set: Historical, gridded population [Internet]. Inter-Sectoral Impact Model Intercomparison Project. 2013 [cited 2017-10-20]. Available from: https://www.isimip.org/gettingstarted/details/31/.
10. CMIP5. Coupled Model Intercomparison Project Phase 5 - Overview. Available from: http://cmip-pcmdi.llnl.gov/cmip5/.
11. Soetaert K, Petzoldt T, Setzer RW. Solving differential equations in R: package deSolve. Journal of Statistical Software. 2010;33.
12. Wikipedia. Bahia 2018. Available from: https://en.wikipedia.org/wiki/Bahia.
13. Worldbank. GDP per capita (current US$) 2018. Available from: https://data.worldbank.org/indicator/NY.GDP.PCAP.CD.
14. Chalom A, Prado P. Multiple runs of the same parameter combination with Rpackage pse.
15. European Centre for Disease Prevention and Control (ECDC). Mosquito maps. *Aedes aegypti & Aedes albopictus* - Current known distribution - January 2016 Stockholm: ECDC; 2016 [cited ECDC. Available from: https://ecdc.europa.eu/en/disease-vectors/surveillance-and-disease-data/mosquito-maps.
16. Hahn MB, Eisen L, McAllister J, Savage HM, Mutebi J-P, Eisen RJ. Updated Reported Distribution of Aedes (Stegomyia) aegypti and Aedes (Stegomyia) albopictus (Diptera: Culicidae) in the United States, 1995–2016. Journal of Medical Entomology. 2017;54(5):1420-4.

17. Marino S, Hogue IB, Ray CJ, Kirschner DE. A Methodology For Performing Global Uncertainty And Sensitivity Analysis In Systems Biology. Journal of theoretical biology. 2008;254(1):178-96.
18. Kraemer MU, Sinka ME, Duda KA, Mylne A, Shearer FM, Brady OJ, et al. The global compendium of Aedes aegypti and Ae. albopictus occurrence. Scientific Data. 2015;2:sdata201535.
